# Epstein–Barr Virus Acquires Its Final Envelope on Intracellular Compartments With Golgi Markers

**DOI:** 10.3389/fmicb.2018.00454

**Published:** 2018-03-16

**Authors:** Asuka Nanbo, Takeshi Noda, Yusuke Ohba

**Affiliations:** ^1^Department of Cell Physiology, Faculty of Medicine and Graduate School of Medicine, Hokkaido University, Sapporo, Japan; ^2^Laboratory of Ultrastructural Virology, Institute for Frontier Life and Medical Sciences, Kyoto University, Kyoto, Japan

**Keywords:** Epstein–Barr virus, final envelopment, egress, *cis*-Golgi, *trans*-Golgi network

## Abstract

Herpesvirus subfamilies typically acquire their final envelope in various cytoplasmic compartments such as the *trans*-Golgi network (TGN), and endosomes prior to their secretion into the extracellular space. However, the sites for the final envelopment of Epstein–Barr virus (EBV), a ubiquitous human gamma herpesvirus, are poorly understood. Here, we characterized the sites for the final envelopment of EBV in Burkitt’s lymphoma cell lines induced into the lytic cycle by crosslinking cell surface IgG. Electron microscopy revealed the various stages of maturation and egress of progeny virions including mature EBV in irregular cytoplasmic vesicles. Immunofluorescence staining showed that gp350/220, the major EBV glycoprotein, and the viral capsid antigen, p18, efficiently colocalized with a *cis*-Golgi marker, GM130. gp350/220 partly colocalized with the TGN, which was distributed in a fragmented and dispersed pattern in the cells induced into the lytic cycle. In contrast, limited colocalization was observed between gp350/220 and endosomal markers, such as a multi-vesicular bodies marker, CD63, a recycling endosome marker, Rab11, and a regulatory secretion vesicles marker, Rab27a. Finally, we observed that treatment of cells with brefeldin A, an inhibitor of vesicle trafficking between the endoplasmic reticulum and Golgi apparatus, resulted in the perinuclear accumulation of gp350/220 and inhibition of its distribution to the plasma membrane. Brefeldin A also inhibited the release of infectious EBV. Taken together, our findings support a model in which EBV acquires its final envelope in intracellular compartments containing markers of Golgi apparatus, providing new insights into how EBV matures.

## Introduction

Epstein–Barr virus (EBV), a ubiquitous human gammaherpesvirus, infects a majority of the population worldwide and establishes a persistent lifelong, mostly asymptomatic infection in them. EBV infection is also associated with various lymphoid and epithelial malignancies such as Burkitt’s lymphoma (BL), Hodgkin’s disease, gastric carcinoma, and nasopharyngeal carcinoma (Longnecker et al., 2013). In latently infected cells the EBV genome is efficiently maintained extrachromosomally (Yates et al., 1996; Nanbo et al., 2007). Either spontaneously or exogenous induction initiates lytic infection, which leads to several 100-fold amplification of the viral genome within 1–2 days (Hammerschmidt and Sugden, 1988). Lytic DNA replication produces long concatemers (Bloss and Sugden, 1994), which eventually are cleaved and packaged as linear genome units in capsids. Replication of EBV DNA and its packaging occur in the nucleus.

Enveloped viruses including herpesvirus acquire host-derived envelope in a variety of intracellular compartments. The three subfamilies of herpesvirus, alphaherpesviruses, betaherpesviruses, and gammaherpesviruses, appear to share a novel mechanism for maturation and egress of virions through several budding and fusion events during replication, which prevents disruption of cellular membranes (Johnson and Baines, 2011; Henaff et al., 2012). This process has been well studied with alphaherpesviruses (Gershon et al., 1994; Zhu et al., 1995; Granzow et al., 1997; Hambleton et al., 2004; Wisner and Johnson, 2004; Sugimoto et al., 2008; Hogue et al., 2014; Buckingham et al., 2016). During a productive infection, viral DNAs are replicated in the nucleus *via* viral machinery. Synthesized viral DNAs are used as templates for gene transcription, which leads to the synthesis of viral structural proteins to produce capsids. Replicated DNAs are then packaged into capsids in the nucleoplasm. Nucleocapsids acquire an envelope by budding through the inner nuclear membranes (INMs) into the perinuclear space. This process is known as the primary envelopment. Perinuclear enveloped virus particles undergo de-envelopment, which is mediated by membrane fusion between their primary envelope and the outer nuclear membrane (ONM). This process delivers nucleocapsids into the cytoplasm, where they acquire a layer of tegument proteins, which ultimately fills the space between the capsids and the envelopes. These tegument proteins promote the earliest stages of herpesvirus replication, including shutting off of host protein synthesis for alphaherpesviruses and transactivation of early viral genes. Tegument-coated nucleocapsids then undergo a secondary envelopment by budding into cytoplasmic compartments, which produce mature virions in these compartments. This process is defined as the final or secondary envelopment. Vesicles containing mature virions are then transported to the cell surface, where they fuse with the plasma membrane (PM) to release virions into extracellular space. For the alphaherpesviruses including herpes simplex virus (HSV), pseudorabies (PRV), and varicella-zoster virus (VZV), the final envelopment occurs in vesicles derived from the *trans*-Golgi network (TGN) (Gershon et al., 1994; Zhu et al., 1995; Granzow et al., 1997; Hambleton et al., 2004; Wisner and Johnson, 2004; Sugimoto et al., 2008; Hogue et al., 2014; Buckingham et al., 2016), and the Rab5-positive early endosomes (EE) (Harley et al., 2001).

Betaherpesviruses such as human cytomegalovirus (HCMV), human herpesvirus type 6 (HHV-6), appear to generate unique compartments by reorganizing pre-existing cellular compartments possessing a variety of cellular markers including TGN, EE, multi-vesicular body (MVB), and late endosome (LE) (Tooze et al., 1993; Sanchez et al., 2000; Fraile-Ramos et al., 2002; Homman-Loudiyi et al., 2003; Das et al., 2007; Mori et al., 2008).

In contrast, the mechanisms of maturation and egress of gammaherpesvirus have been poorly characterized.

In some previous studies, electron microscopic analysis visualized the various stages of the life-cycles of EBV (Seigneurin et al., 1977; Greenspan et al., 1989; Lee and Longnecker, 1997; Lake and Hutt-Fletcher, 2000) and of Kaposi sarcoma-associated herpesvirus (KSHV) (Orenstein et al., 1997). Moreover, Peng et al. (2010) took advantage of electron tomography to characterize the ultrastructural basis of the MHV-68 lifecycle. They showed that tegumented capsids bud into vesicles located adjacent to Golgi apparatus to acquire a final envelope. These findings suggest that EBV shares the mechanism of virion maturation and egress with other herpesvirus subfamilies, however, the sites for its final envelopment have not been demonstrated because of the lack of a fully permissive culture model for the lytic cycle. Some latently EBV-infected BL cell lines can be induced into the lytic cycle with varying efficiency by treatment with chemical inducers, such as phorbol esters, *n*-butyrate, or by ligation of surface immunoglobulin (zur Hausen and Fresen, 1978; Takada, 1984; Faggioni et al., 1986).

Here, we have characterized the sites for the final envelopment of EBV in the Akata BL cell line (Takada et al., 1991; Shimizu et al., 1994; Nanbo et al., 2002, 2012, 2013, 2016) induced into the lytic cycle by crosslinking of surface immunoglobulin. Akata cells are particularly responsive to this treatment, with up to 50% of cells converting to the lytic form of infection in a synchronous manner.

Our findings demonstrate that the final envelopment of EBV takes place in compartments containing *cis-*Golgi and TGN characteristics. We also evaluate a mechanism for the maturation and egress of EBV virions based on these findings.

## Materials and Methods

### Cell Culture

Epstein–Barr virus-positive African BL-derived Akata (Akata^+^) cells (Takada et al., 1991; Shimizu et al., 1994; Nanbo et al., 2002, 2012, 2013, 2016) were maintained in RPMI-1640 medium containing 10% fetal bovine serum (FBS) (Sigma-Aldrich, St. Louis, MO, United States) and antibiotics. EBV-negative BL Daudi (Daudi^-^) cells were isolated from EBV-positive clones by the limiting dilution (Klein et al., 1968). Akata^-^ EBV-eGFP cells are latently infected with a recombinant Akata strain EBV encoding eGFP gene inserted into viral BXLF1 ORF (EBV-eGFP) (Maruo et al., 2001a; Nanbo et al., 2012, 2016). Akata^-^ EBV-eGFP cells were maintained in RPMI-1640 medium containing 10% FBS, antibiotics and 800 μg/ml G418 (Wako Pure Chemical Industries Ltd., Osaka, Japan). Cells were maintained at 37°C in 5% CO_2_. For induction of the lytic cycle of EBV, Akata^+^ cells or Akata^-^ EBV-eGFP cells were treated with 1% goat anti-human IgG (αhIgG; Dako, Glostrup, Denmark) (Takada, 1984; Takada and Ono, 1989).

### Electron Microscope

Ultrathin-section electron microscopy was performed as previously reported (Noda et al., 2002, 2006). Briefly, 36 h after αhIgG treatment, Akata^-^ EBV-eGFP were washed with PBS and fixed with 2.5% glutaraldehyde in 0.1 M cacodylate buffer (pH 7.4) for 1 h at 4°C. Small pieces of fixed pellet were washed with the same buffer, post fixed with 2% osmium tetroxide in the same buffer for 1 h at 4°C, dehydrated with a graded series of ethanol concentrations followed by propylene oxide, embedded in Epon 812 Resin mixture (TAAB Laboratories Equipment Ltd., Berks, England), and polymerized at 70°C for 2 days. Thin sections were stained with uranyl acetate and lead citrate and examined with a Hitachi H-7650 electron microscope (Hitachi Ltd., Japan) at 80 kV.

### Immunofluorescence Staining

Akata^+^ cells (2 × 10^6^ cells) were treated with 1% αhIgG for 16 h in 6-well plate. The cells were fixed with 4% paraformaldehyde (PFA) in PBS for 10 min at room temperature (r.t.), permeabilized with PBS containing 0.05% Triton X-100 for 10 min at r.t., and blocked in PBS containing 1% bovine serum albumin (BSA) for 20 min at r.t. The cells were incubated with mouse anti-EBV gp350/220 monoclonal antibody (clone C-1, 1:200 dilution) (Thorley-Lawson and Geilinger, 1980), goat anti-EBV p18 polyclonal antibody (Thermo Fisher Scientific, Waltham, MA, United States, 1:200 dilution), rabbit anti-TGN64 (Novus Biologicals, Littleton, CO, United States, 1:200 dilution), rabbit anti-GM130 monoclonal antibody (Cell Signaling Technology, clone D6B1, 1:200 dilution), rabbit anti-CD63 polyclonal antibody (Abcam, Cambridge, United Kingdom, 1:200 dilution), rabbit anti-Rab27a polyclonal antibody (Abcam, 1:200 dilution), or anti-Rab11 polyclonal antibody (Abcam, 1:200 dilution) for 1 h at r.t. After washing twice in PBS, the cells were incubated with Alexa Fluor 488, 594, or 647-labeled secondary antibodies (Thermo Fisher Scientific, 1:1000 dilution) for 1 h at room temperature. After washing twice in PBS, the nuclei were counterstained with Hoechst 33342 (Cell Signaling Technology, Beverly, MA, United States). Images were collected with a 60× water-immersion objective (NA = 1.3) of a confocal laser scanning microscope (Fluoview FV10i, Olympus, Tokyo, Japan) and acquired by using FV10-ASW software (Olympus). Line scan imaging was performed by using FV10-ASW software.

### Western Blotting

Induction of lytic cycle was confirmed by Western blotting by use of goat anti-EBV p18 polyclonal antibody (Thermo Fisher Scientific, 1:1000 dilution). The effect of induction of lytic cycle of EBV on the expression of organelle markers was analyzed by Western blot analysis with rabbit anti-TGN64 (1:500 dilution), rabbit anti-GM130 monoclonal antibody (1:1000 dilution), rabbit anti-CD63 polyclonal antibody (Wako pure chemical industries Ltd., 1:1000 dilution), mouse anti-Rab11 monoclonal antibody (BD Biosciences, San Jose, CA, United States, 1:1000 dilution), or rabbit anti-Rab27a polyclonal antibody (Sigma-Aldrich, 1:500 dilution).

### Brefeldin A Treatment

For brefeldin A (BFA) treatment, 100 nM BFA (Cell Signaling Technology) was added to Akata^+^ cells (5 × 10^5^ cells) at 6 h post-induction of the lytic cycle. After 10 h, the cells were harvested, and immunofluorescence staining was performed. To assess the effect of BFA on EBV egress, 100 nM BFA was added to Akata^-^ eGFP-EBV cells at 6 h post-induction of the lytic cycle. Two days post-induction, the supernatants containing eGFP-EBV were harvested and incubated with Daudi^-^ cells (2 × 10^5^ cells) in 24-well plate for 1 h at 37°C. After being washed the cells were incubated for 48 h later, the percentage of eGFP-positive infected cells was measured by flow cytometry (FACSCalibur, Becton, Dickinson and Company). The effect of BFA on the synthesis of EBV-encoded p18 was confirmed by Western blot analysis.

## Results

### EBV Capsids Acquire Their Primary Envelopment by Sequential Budding and Fusion Through the Perinuclear Cisterna

To characterize the site for the final envelopment of EBV, the viral lytic cycle was induced by cross linking the cell surface IgG of Akata^-^ EBV-eGFP by adding F(ab’)_2_ fragments of goat anti-human IgG polyclonal antibody (αhIgG) (Takada, 1984).

**FIGURE 1:**
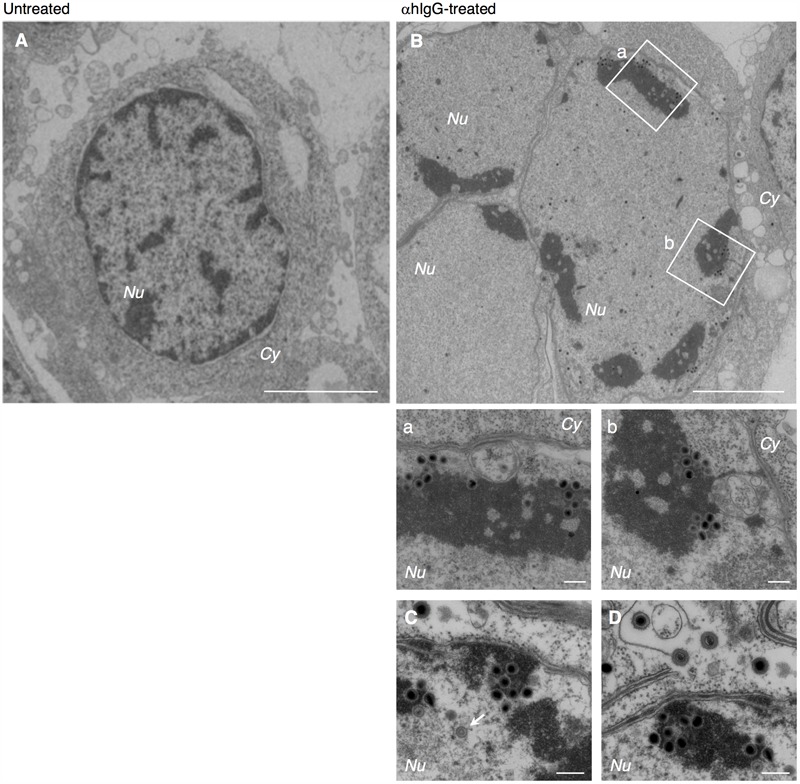
Electron micrograph showing nucleocapsid formation in the nucleus of Akata cells induced into the lytic cycle of EBV. Morphological features of Burkitt’s lymphoma cells induced lytic infection of EBV. Akata^-^ eGFP-EBV cells were treated without **(A)** or with **(B)** αhIgG for 24 h. Low **(A,B)** and high **(a,b,C,D)** power views of Akata^-^ eGFP-EBV cells. **(a)** and **(b)** represent boxed areas in **(B)**. The white arrow indicates a double-layered capsid without core. Scale bars: 2.5 μm **(A,B)** and 250 nm **(a,b,C,D)**. In all the electron micrographs, italic abbreviations used for describing cellular compartments are as follows; Cy, cytoplasm; Nu, nucleus; Ve, vesicles; Ex, extracellular space; PM, plasma membrane; INM, inner nuclear membrane; ONM, outer nuclear membrane.

The cells were harvested after treatment with αhIgG for 36 h and the intracellular distribution of progeny virions were analyzed by electron microscopy. Consistent with previous reports, we observed multiple capsids containing the electron-dense cores inside in the nucleus of infected cells (**Figures 1A** vs. **1B**). Capsids were frequently associated with less electron-dense materials than DNA cores (**Figures 1B–D**), which appeared to be replication compartments. We also observed a double-layered capsid without a core in the specimen (arrow in **Figure 1C**), which is likely a transient intermediate of capsid assembly.

**FIGURE 2:**
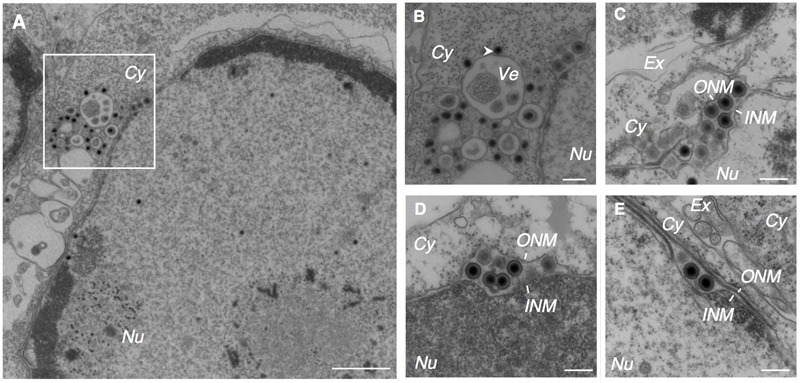
EBV acquires its primary envelope by budding into the perinuclear space. Morphological features of Akata^-^ eGFP-EBV cells induced into the lytic cycle showing primary envelopment and nuclear egress. Akata^-^ eGFP-EBV cells were treated with αhIgG for 24 h. Low **(A)** and high **(B–E)** power views of Akata^-^ eGFP-EBV cells. **(B)** Represents boxed area in **(A)**. The white arrowhead indicates high electron-dense material. Scale bars: 1 μm **(A)** and 250 nm **(B–E)**.

**FIGURE 3:**
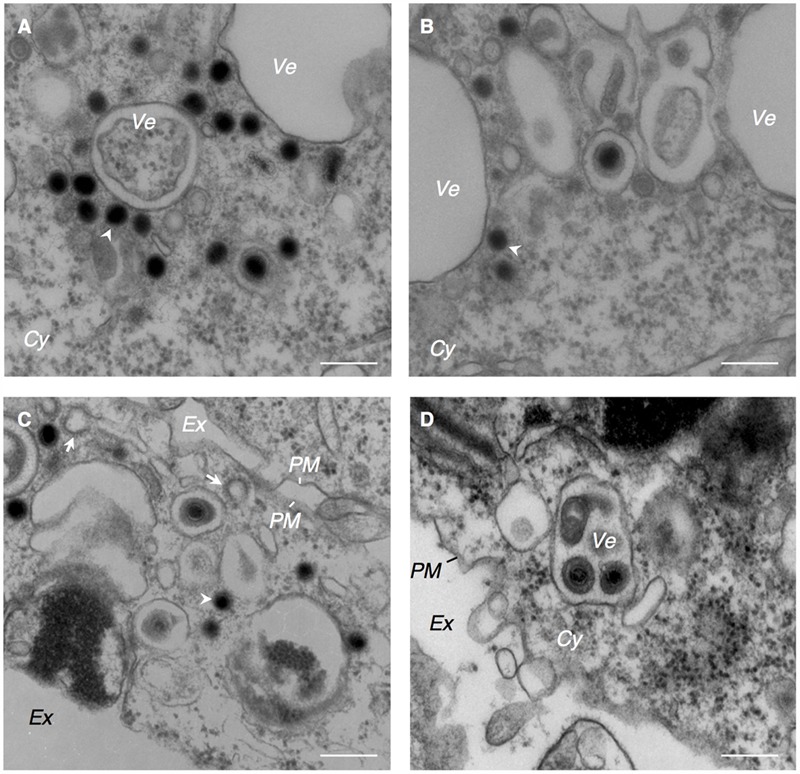
EBV capsids acquire final envelopes in the cytoplasmic compartments. Morphological features of Akata^-^ eGFP-EBV cells induced into lytic cycle showing acquisition of secondary envelope of EBV. Akata^-^ eGFP-EBV cells were treated with αhIgG for 24 h. High power views of Akata^-^ eGFP-EBV cells **(A–D)**. The white arrowheads indicate high electron-dense material. The white arrows indicate the clathrin-coated vesicles. Scale bars: 250 nm.

We found that multiple irregular vesicles were formed in the cytoplasm in the lytically induced cells (**Figures 1B, 2A**). We also detected enveloped capsids in the perinuclear space between the INM and the ONM (**Figure 2**), suggesting that mature capsids bud into the perinuclear space to acquire a primary envelope. The envelopes were smooth without protrusions on their surface. In addition, the capsids that lack envelopes were distributed in the cytoplasm (**Figures 2A, 3A–C**), indicating that fusion between the primary envelopment and the ONM serves as a de-envelopment process, resulting in the release of capsids lacking envelope into the cytoplasm. These results confirmed the model that EBV acquires its primary envelopment by sequentially budding and fusion through the perinuclear cisterna as demonstrated previously (Seigneurin et al., 1977; Lee and Longnecker, 1997; Peng et al., 2010).

### EBV Capsids Acquire Tegument and Undergo a Final Envelopment in the Cytoplasm

**FIGURE 4:**
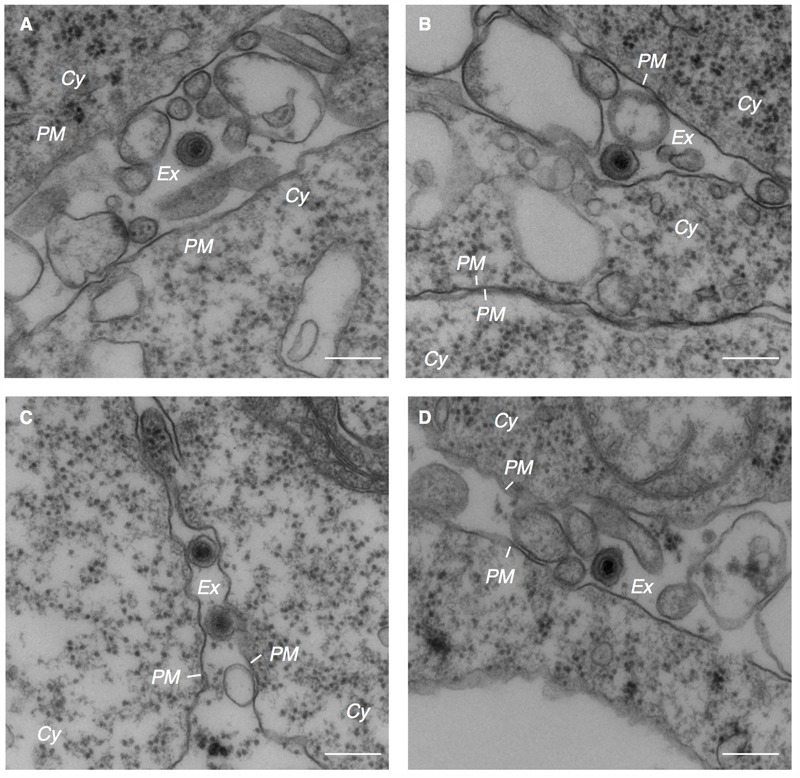
Electron micrograph showing extracellular mature virions. Extracellular mature virions of EBV. Akata^-^ eGFP-EBV cells were treated with αhIgG for 24 h. High power views of Akata^-^ eGFP-EBV cells **(A–D)**. Scale bars: 250 nm.

The cytoplasmic capsids were surrounded by electron-dense materials which appeared to be derived from tegument proteins (arrowheads in **Figures 2B, 3A–C**). This process occurred in the vicinity of cytoplasmic vesicles of various sizes that contained one or multiple enveloped capsids possessing tegument material. Spike-like protrusions attributable to glycoproteins were present on the envelopes. Vesicles containing mature virions were observed in the vicinity of the PM (**Figure 3D**). Several extracellular mature virions were observed in the peripheral region of the PM (**Figure 4**). They were composed of a bilayer envelope with spikes, tegument materials in between bilayer envelope and a DNA core. All these results indicate that the egress of EBV likely take places *via* the exocytosis pathway, consistent with the model favored for other herpesviruses.

### EBV Structural Proteins Distribute in Compartments Containing *Cis-* and *Trans*-Golgi Markers

Consistent with the notion of secondary envelopment of other herpesvirus subfamilies (Campadelli et al., 1993; Zhu et al., 1995; Das et al., 2007; Mori et al., 2008; Sugimoto et al., 2008; Cepeda et al., 2010; Hogue et al., 2014; Buckingham et al., 2016), EBV has been thought to exploit TGN- and endosome-derived vesicles to acquire its final envelopment. We observed that clathrin-coated vesicles, which originate from the Golgi complex (Robinson and Bonifacino, 2001), localized adjacent to the vesicles containing capsids (arrows in **Figure 3C**), although intact Golgi structures were not observed in the analyzed specimens.

**FIGURE 5:**
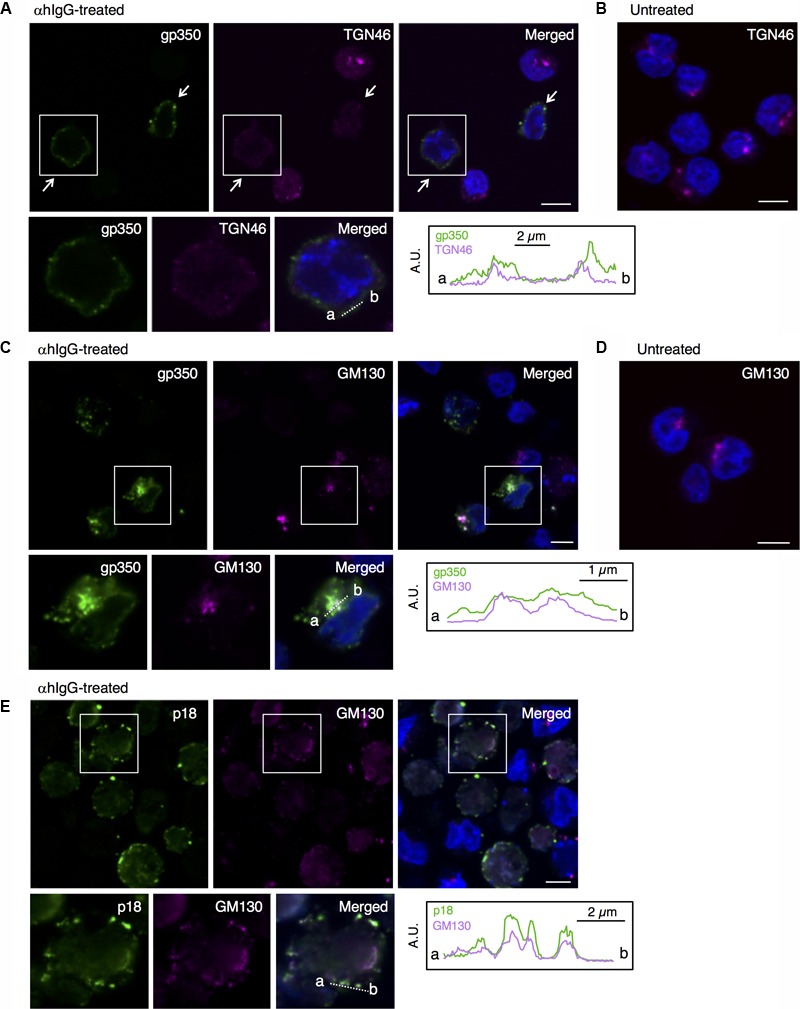
EBV structural proteins distribute in the compartments containing *cis-*Golgi and the TGN markers. The distribution of the EBV structural proteins and markers of Golgi apparatus in Akata^+^ cells induced into the lytic cycle. Akata^+^ cells were treated with αhIgG **(A,C,E)** or without **(B,D)** for 16 h. **(A)** The distribution of gp350/220 (left), TGN64 (middle) and merged (right) images are shown. As a control, the distribution of TGN64 in the untreated cells is shown in **(B)**. **(C)** The distribution of gp350/220 (left), GM130 (middle) and merged (right) are shown. As a control, the distribution of GM130 in the untreated cells is shown in **(D)**. **(E)** The distribution of the EBV capsid antigen, p18 (left) and GM130 (middle) and merged (right) images are shown. The nuclei (blue) were counterstained with Hoechst 33342. Insets show the boxed areas. The plots indicate the individual fluorescence intensity along each of the corresponding lines. A.U., arbitrary unit. Scale bars: 10 μm.

We then characterized from which Golgi subcompartments EBV acquires its final envelope by immunofluorescence staining. We first assessed the distribution of one of major viral envelope glycoprotein, gp350/220 (Nemerow et al., 1987; Maruo et al., 2001b), which is expressed during the late phase of the lytic cycle, by immunofluorescence staining. At 16 h post-induction of the lytic cycle, gp350/220 distributed in the cytoplasm and the PM in a speckled pattern (green in **Figure 5A**). We further analyzed the distribution of TGN46, which is generally used as a marker of TGN. TGN46 distributed in the perinuclear region in mock-infected cells (**Figure 5B**). On the other hand, TGN46 localized in a dispersed pattern in the cells expressing gp350/220 and limited signals were observed (arrows in **Figure 5A**). Western blot analysis revealed that TGN46 expression was partly down-regulated in Akata cells treated with αhIgG (**Figure 7**). gp350/220 partly colocalized with TGN46. We assessed the distribution of *cis-*Golgi marker, GM130 in the cells induced lytic cycle. As expected, GM130 distributed in the perinuclear region in mock-infected cells (**Figure 5D**). Unlike TGN46, the distribution of GM130 was largely unperturbed in gp350/220-positive cells (magenta in **Figures 5C, 7**). Moreover, gp350/220 (green) efficiently colocalized with GM130 signals (**Figure 5C**). To confirm that mature virions bud into the GM130-positive cellular compartments, we examined the distribution of GM130 and the EBV viral capsid antigen (VCA)-p18, BFRF3 (van Grunsven et al., 1994; Henson et al., 2009). VCA-p18 distributed in the nucleus diffusely, and in the cytoplasm and the PM in a speckled pattern (green in **Figure 5E**). GM130 efficiently colocalized with p18 in the cytoplasm and in the PM (magenta in **Figure 5E**). These results indicate that secondary envelopment of EBV takes place in the intracellular compartments containing *cis-* and TGN markers.

### EBV Structural Proteins Do Not Colocalize With Secretory Endosomal Markers

**FIGURE 6:**
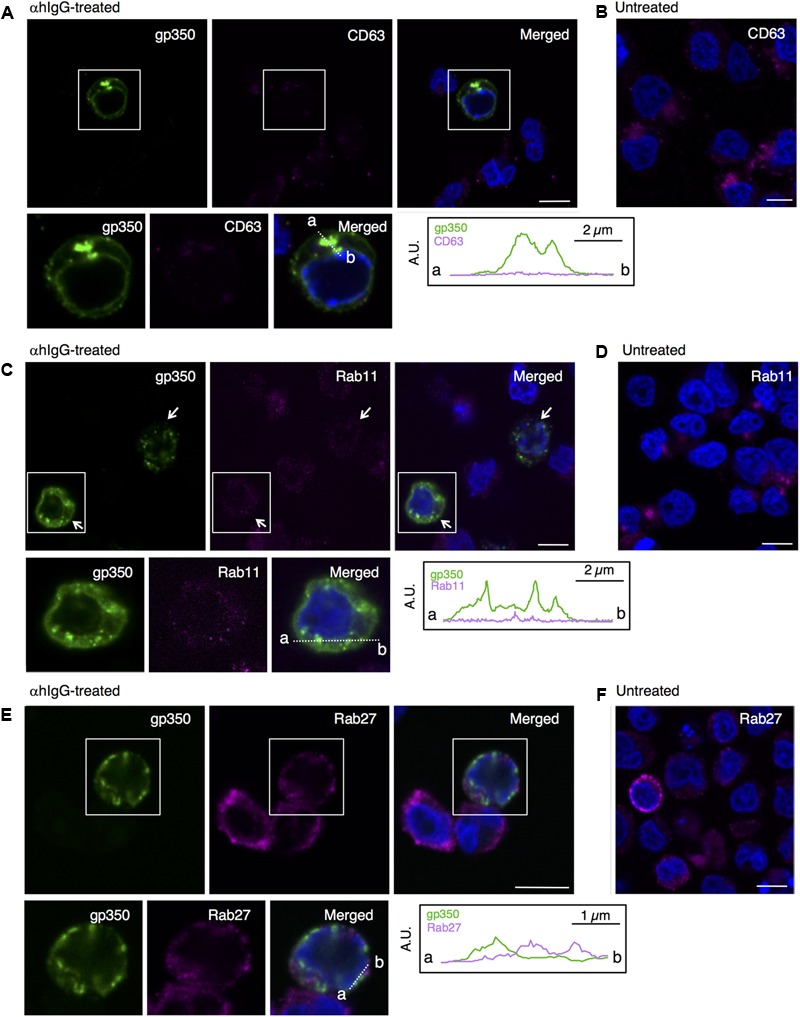
EBV glycoprotein does not colocalize with endosomal markers. The distribution of the EBV glycoprotein and markers of endosomes in Akata^+^ cells induced into the lytic cycle. Akata^+^ cells were treated with **(A,C,E)** or without αhIgG **(B,D,F)** for 16 h. **(A)** The distribution of gp350/220 (left), CD63 (middle) and merged (right) images are shown. As a control, the distribution of CD63 in the untreated cells is shown in **(B)**. **(C)** The distribution of gp350/220 (left), Rab11 (middle) and merged (right) are shown. As a control, the distribution of Rab11 in the untreated cells is shown in **(D)**. **(E)** The distribution of the EBV capsid antigen, gp350/220 (left) and Rab27a (middle) and merged (right) images are shown. As a control, the distribution of Rab27a in the untreated cells is shown in **(F)**. The nuclei (blue) were counterstained with Hoechst 33342. Insets show the boxed areas. The plots indicate the individual fluorescence intensity along each of the corresponding lines. A.U., arbitrary unit. Scale bars: 10 μm.

**FIGURE 7:**
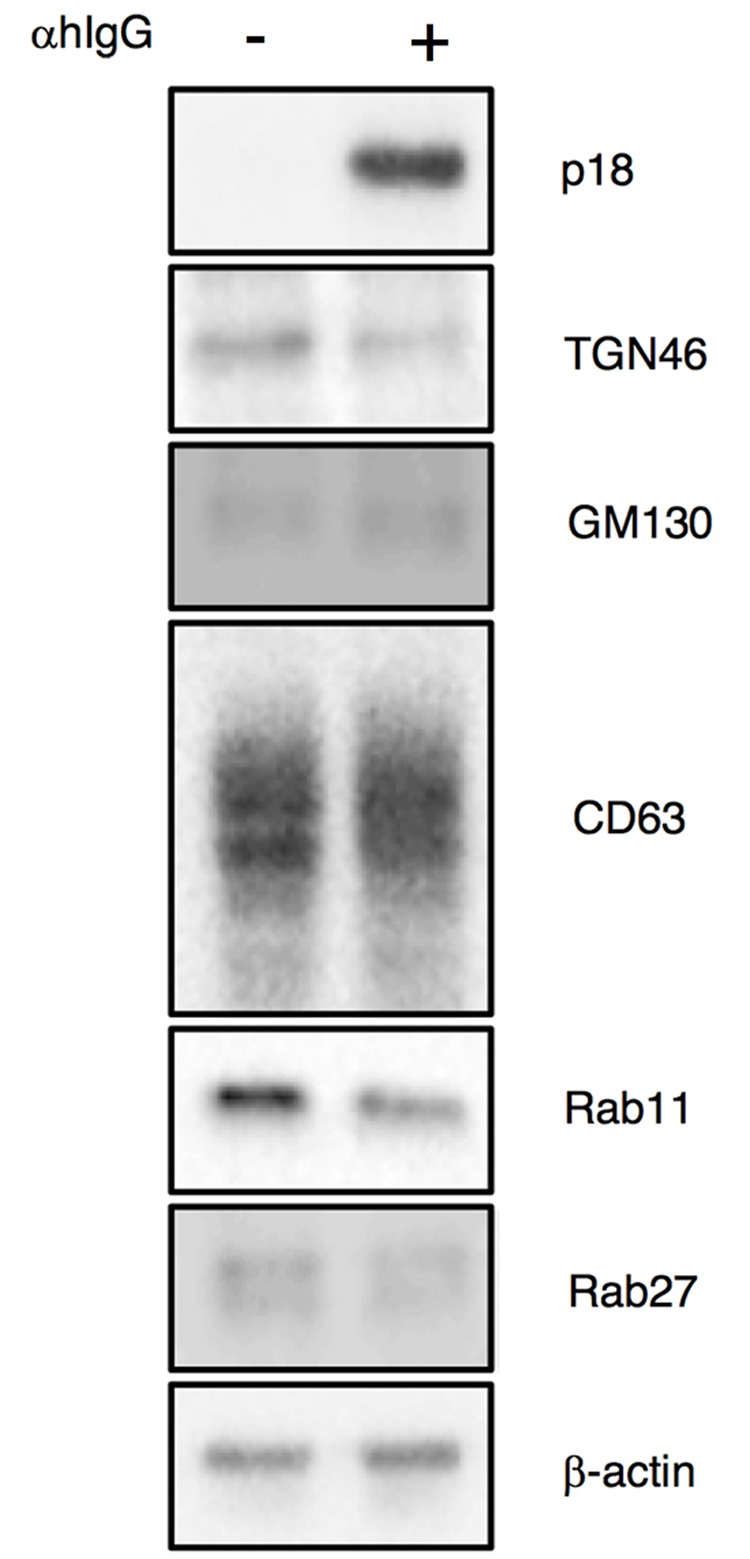
Expression of organelle markers in Akata^+^ cells treated with αhIgG. Akata^+^ cells were treated with or without αhIgG for 16 h. Total cell lysates were analyzed by Western blotting with antibodies against p18, TGN46, GM130, CD63, Rab11, Rab27a, or β-actin.

Because alphaherpesvirus and betaherpesvirus subfamilies acquire secondary envelopes by budding into the vesicles containing endosomal markers (Bloss and Sugden, 1994; Wisner and Johnson, 2004; Das et al., 2007; Mori et al., 2008; Sugimoto et al., 2008; Cepeda et al., 2010; Hogue et al., 2014), we further investigated whether gp350/220 distributes in endosomes. We first examined the distribution of a marker for MVB, tetraspanin protein CD63 (van Niel et al., 2011). While CD63 distributes in the perinuclear region in control cells (**Figure 6B**), CD63 was visualized as speckles in the cells treated with αhIgG (magenta in **Figure 6A**). gp350/220 (green) did not efficiently colocalized with CD63 signals (**Figure 6A**). We next assessed the role of recycling endosomes as a site for EBV maturation by use of a marker, Rab11 (Wilcke et al., 2000; Takahashi et al., 2012). Consistent with CD63, Rab11 was detected in the perinuclear region in untreated cells (**Figure 6D**). Upon induction of the lytic cycle, Rab11 signals (magenta) decreased and scattered in gp350/220-positive cells (arrows in **Figure 6C**). Down-regulation of Rab11 expression in αhIgG-treated Akata cells was also determined by Western blotting (**Figure 7**). No efficient colocalization was observed between Rab11 and gp350/220. Finally, we tested a small GTPase, Rab27a, which is associated with lysosome-related organelles (Neeft et al., 2005) and involved in regulation of the secretion pathway (Ostrowski et al., 2010; Zografou et al., 2012). Rab27a distributed in a speckled pattern in the cytoplasm and its expression level was heterogeneous in the cell population (**Figure 6F**). We observed the gp350/220 (green) did not efficiently colocalize with the Rab27a-signal (magenta in **Figure 6E**). Taken together, it is unlikely that EBV capsids acquire the final envelope in endosomal compartments.

### Brefeldin A Treatment Induces the Intracellular Accumulation of EBV Glycoproteins

**FIGURE 8:**
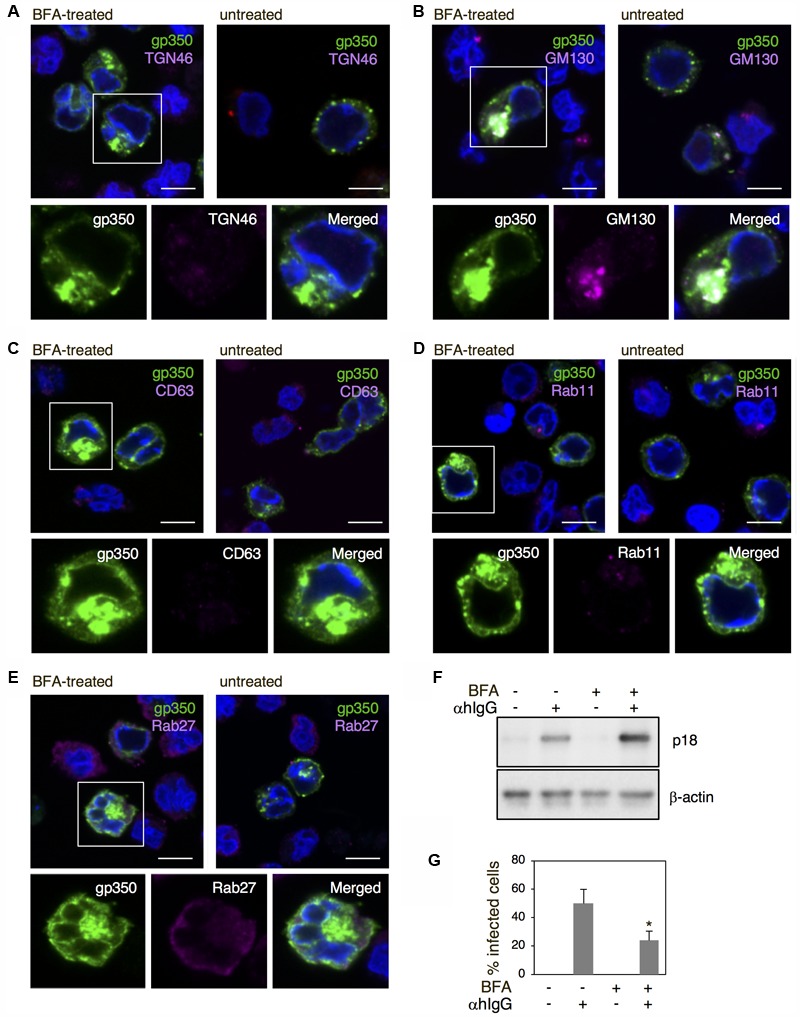
The effect of BFA on the distribution of EBV structural proteins and the release of virions. **(A–E)** The effect of BFA on the distribution of gp350/220 and organelle markers in Akata^+^ cells induced into the lytic cycle. Akata^+^ cells were treated with αhIgG in the presence or absence of 100 nM BFA for 16 h. The distribution of gp350/220 (green), TGN46 **(A)**, GM130 **(B)**, CD63 **(C)**, Rab11 **(D)**, and Rab27a **(E)** are shown. The nuclei were counterstained with Hoechst 33342. Insets show the boxed areas. Scale bars: 10 μm. **(F)** The effect of BFA on expression of p18 in Akata^+^ cells undergoing the lytic cycle. Akata^+^ cells were treated with or without αhIgG in the presence or absence of 100 nM BFA for 16 h. Total cell lysates were analyzed by Western blotting with antibodies against p18 or β-actin. **(G)** The effect of BFA on the release of virions. Akata^-^ eGFP-EBV cells were treated with or without αhIgG in the presence or absence of 100 nM BFA for 48 h. Supernatants containing eGFP-EBV were harvested and incubated with Daudi^-^ cells for 24 h. The percentages of eGFP-positive infected Daudi^-^ cells were analyzed by means of flow cytometry. The experiment was performed three times independently and the average values and their standard deviations are shown for each condition. ^∗^*P* < 0.05 versus respective control (Student’s *t*-test).

We further assessed the role of the Golgi apparatus in the final envelopment of EBV by means of brefeldin A (BFA), a metabolite of the fungus *Eupenicillium brefeldianum*. BFA specifically and reversibly blocks protein transport from the ER to the Golgi apparatus (Lippincott-Schwartz et al., 1989; Pelham, 1991; Hunziker et al., 1992), resulting in a blockage of vesicular assembly, antigen presentation, transcytosis, endocytosis, and viral assembly and budding (Cheung et al., 1991; Whealy et al., 1991; Chatterjee and Sarkar, 1992). The lytic cycle was induced in the presence of 100 nM BFA and the distribution of gp350/220 and cell organelle markers were analyzed by immunofluorescence staining. In contrast to untreated cells, treatment with BFA induced an accumulation of gp350/220 in the perinuclear region (green in **Figures 8A–E**). Furthermore, gp350/220 was not detectable in the PM. TGN46 distributed diffusely in αhIgG-treated cells in the presence of BFA and did not associate detectably with gp350/220 (**Figure 8A**). Signals from gp350/220 often colocalized with GM130 (**Figure 8B**). In contrast, gp350/220 did not colocalize with CD63, Rab11, and Rab27a (**Figures 8C–E**). These results indicate that BFA blocks transport of gp350/220 to the Golgi apparatus and its subsequent trafficking to the PM. We also measured the effect of BFA on the release of infectious virions. Akata^-^ eGFP-EBV cells were induced into the lytic cycle in the presence of BFA. Two days post-induction, the supernatant containing eGFP-EBV was harvested and incubated with EBV-negative BL-derived Daudi^-^ cells. Forty-eight hours later, the percentage of eGFP-positive, infected cells was analyzed by flow cytometry. We found that BFA significantly inhibited the release of matured virions (**Figure 8G**). BFA treatment increased expression of p18 in the lytic cycle-induced cells (**Figure 8F**), suggesting the intracellular accumulation of gp350/220. These results demonstrate that the final envelopment is established on the Golgi apparatus followed by egress though an unknown exocytotic machinery.

## Discussion

Accumulating evidence indicates that herpesvirus subfamilies likely share a mechanism for maturation and egress of their progeny virions (Johnson and Baines, 2011; Henaff et al., 2012). However, the mechanism underlying the acquisition of the final envelopment of gammaherpesvirus is poorly understood.

Previous studies characterized viral genes that are responsible for the primary envelopment of EBV. The EBV BGLF4 kinase modified the structure of nuclear lamina to initiate the egress of nucleocapsids (Gershburg et al., 2007; Lee et al., 2008). BFRF1 and BFLF2, which are highly conserved homologs among the herpesvirus family, have been shown to be involved in the nuclear egress of EBV. Moreover, BFRF1 exploits the host endosomal sorting complex required for transport (ESCRT) machinery to induce the reorganization of nuclear membrane followed by efficient nuclear egress (Gonnella et al., 2005; Lee et al., 2012). In contrast, the mechanism for final envelopment of EBV has remained unclear because of the lack of an efficient viral replication model.

Here, we characterized the sites for the final envelopment of EBV in BL-derived Akata cells induced into the lytic cycle by crosslinking cell surface IgG (Takada, 1984). Electron microscopic analysis visualized the formation of nucleocapsids in the nucleus (**Figure 1**), egress of enveloped nucleocapsids into the perinuclear space (**Figure 2**), release of cytoplasmic nucleocapsid lacking envelope (**Figure 3**), and irregular cytoplasmic vesicles containing mature virions (**Figure 3**). These results support a model in which EBV matures via a similar pathway to other herpesviruses, as previously thought (Johnson and Baines, 2011; Henaff et al., 2012).

**FIGURE 9:**
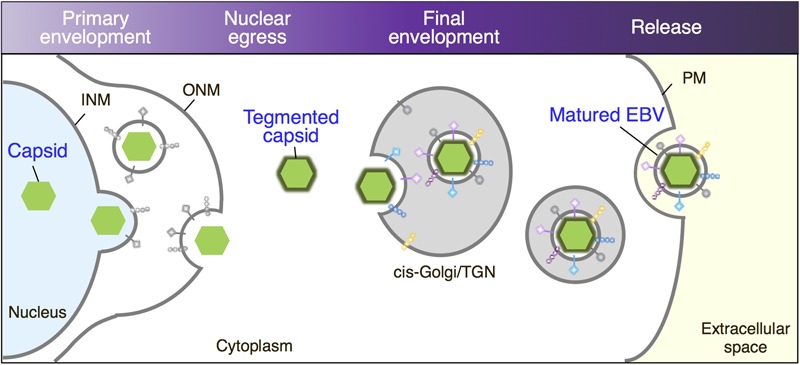
Maturation of EBV virions. Replicated viral DNAs are packaged into capsids in the nucleoplasm. Nucleocapsids acquire primary envelopes by budding through the INM into the perinuclear space. Perinuclear enveloped virus particles undergo de-envelopment, which is mediated by membrane fusion between their primary envelope and the ONM (Nuclear egress). Tegument-coated nucleocapsids then undergo a final envelopment by budding into intracellular compartments derived from *cis-*Golgi/TGN, which produce mature virions in these compartments. Vesicles containing mature virions are then transported to the cell surface, fused with the PM to release virions into extracellular space.

We further characterized the origins of the vesicles in which mature viruses bud and found that Golgi apparatus markers such as GM130 and TGN46 colocalize with the viral major glycoprotein gp350/220 and the VCA p18 (**Figures 5A,C,E**), suggesting that the final envelopment site for EBV originates from the Golgi apparatus as illustrated in **Figure 9**.

We often observed fragmented and dispersed TGN46 signals in the cytoplasm and periphery of the cells expressing EBV structural proteins (**Figures 5A** vs. **5B**). HSV-1 infection also induces a similar distribution of TGN and endosomal compartments to the PM and cell-to-cell junctions, suggesting that it may reflect disruption of balanced bi-directional ER-Golgi transport induced by abnormal influx of viral glycoproteins and/or reorganization of TGN compartments to mediate virus egress by an exocytic pathway (Campadelli et al., 1993; Wisner and Johnson, 2004). In contrast, a limited alteration of the morphology and distribution of *cis-*Golgi was observed (**Figures 5C** vs. **5D**). Since BFA treatment induced the accumulation of gp350/220 in the perinuclear region (**Figure 8**), EBV appears to target GM130-positive vesicles to provide the final envelopment, followed by subsequent trafficking through TGN to the PM for viral egress.

We observed that electron-dense materials were associated with cytoplasmic capsids lacking envelope in the vicinity of cytoplasmic vesicles (**Figures 2, 3**).

It has been shown that herpesvirus tegument proteins possess sorting signals for specific intracellular compartments, where the viral glycoproteins are loaded. The interaction between tegument proteins, membrane proteins, and glycoproteins in cytoplasmic compartments is crucial for the final envelopment (Johnson and Baines, 2011; Henaff et al., 2012). The distribution of limited tegument proteins has been revealed for EBV.

BNRF1, the EBV major tegument protein, which is encoded only by members of the gammaherpesvirus subfamily, distributes both in the cytoplasm and nucleus (Tsai et al., 2011). While BNRF1 has been shown to localize in PML nuclear bodies, its cytoplasmic distribution has not been studied in detail. Another tegument protein BBLF1, a homolog of UL11 for HSV-1 and UL99 for HCMV, has been shown to be involved in the final envelopment. Both endogenous and exogenously expressed BBLF1 are predominantly colocalized with TGN46. In contrast, overexpressed BBLF1 partially colocalized with GM130. BBLF1 did not colocalize with EEA1 and LAMP2, an early endosome and a late endosome marker, respectively (Chiu et al., 2012). Exogenously expressed BRRF2 gene, which is also conserved only in gammaherpesvirus subfamily members such as KSHV and MHV-68, partly localized with Rab5 but not with the ER and the Golgi apparatus markers (Watanabe et al., 2017). Because any inconsistency in the distribution of these tegument proteins might reflect the overexpression of protein alone, a further characterization of individual tegument proteins is essential in cells induced into EBV’s lytic cycle.

Unlike alphaherpesviruses, betaherpesviruses are known to induce their own compartment containing various organelle markers (Tooze et al., 1993; Sanchez et al., 2000; Fraile-Ramos et al., 2002; Homman-Loudiyi et al., 2003; Das et al., 2007; Mori et al., 2008). We observed that irregularcytoplasmic vesicles are formed in lytically induced B cells (**Figures 1B, 2A, 3**), suggesting that EBV also reorganizes the pre-existing intracellular compartment to initiate secondary envelopment sites. A recent study has demonstrated that macroautophagic membranes are stabilized by the lytic cycle of EBV and macroautophagic proteins are incorporated into EBV virions (Nowag et al., 2014), suggesting that EBV exploits various cellular machinery for its efficient envelope acquisition.

The vesicles containing mature EBV subsequently traffic to the PM and release virions extracellularly (**Figure 4**). In this study, viral structural proteins were not efficiently colocalized with endosomal markers, such as CD63, Rab11, and Rab27a, which are responsible for secondary envelopment and the egress of other herpesvirus subfamilies (Bloss and Sugden, 1994; Wisner and Johnson, 2004; Das et al., 2007; Mori et al., 2008; Sugimoto et al., 2008; Cepeda et al., 2010; Hogue et al., 2014). We found that signals of some organelle markers such as TGN46 and Rab11 decreased in the cells expressing viral lytic genes (**Figures 5A, 6C, 7**). In a previous study, mature EBV was found to incorporate viral and host proteins identified by tandem mass spectrometry (Johannsen et al., 2004). Limited organelle markers, such as clathrin-heavy chain and Rab1A were identified in these preparations. These findings indicate that the lytic cycle of EBV down-regulates expression of particular host genes, which may interfere with the analysis of EBV structural proteins colocalizing with organelle markers. To understand the detailed mechanism of EBV’s maturation and egress, further investigations by use of alternative methods such as the immune electron microscopy and super resolution microscopy are required.

Taken together, our findings support a model in which EBV acquires its final envelope in intracellular compartments containing markers of Golgi apparatus. These findings provide new insights into our understanding of the mechanism of EBV’s maturation.

## Author Contributions

AN was involved in conceptualizing the study, experimental design, and data analysis. AN and TN conducted the experiments. AN wrote the draft that was edited by TN and YO.

## Conflict of Interest Statement

The authors declare that the research was conducted in the absence of any commercial or financial relationships that could be construed as a potential conflict of interest.
